# Multitier Web System Reliability: Identifying Causative Metrics and Analyzing Performance Anomaly Using a Regression Model

**DOI:** 10.3390/s23041919

**Published:** 2023-02-08

**Authors:** Sundeuk Kim, Jong Seon Kim, Hoh Peter In

**Affiliations:** 1 Department of Computer Science, Korea University, Seoul 02841, Republic of Korea; 2 Platform Planning Group, Samsung SDS, Seoul 05510, Republic of Korea; 3 MSP Development Group, Samsung SDS, Seoul 05510, Republic of Korea

**Keywords:** anomaly detection, causative metrics, regression model, distributed systems

## Abstract

With the development of the Internet and communication technologies, the types of services provided by multitier Web systems are becoming more diverse and complex compared to those of the past. Ensuring a continuous availability of business services is crucial for multitier Web system providers, as service performance issues immediately affect customer experience and satisfaction. Large companies attempt to monitor the system performance indicator (SPI) that summarizes the status of multitier Web systems to detect performance anomalies at an early stage. However, the current anomaly detection methods are designed to monitor a single specific SPI. Moreover, the existing approaches consider performance anomaly detection and its root cause analysis separately, thereby aggravating the burden of resolving the performance anomaly. To support the system provider in diagnosing the performance anomaly, we propose an advanced causative metric analysis (ACMA) framework. First, we draw out 191 performance metrics (PMs) closely related to the target SPI. Among these PMs, the ACMA determines 62 vital PMs that have the most influence on the variance of the target SPI using several statistical methods. Then, we implement a performance anomaly detection model to identify the causative metrics (CMs) between the vital PMs using random forest regression. Even if the target SPI changes, our detection model does not require any change in its model structure and can derive closely related PMs of the target SPI. Based on our experiments, wherein we applied the ACMA to the business services in an enterprise system, we observed that the proposed ACMA could correctly detect various performance anomalies and their CMs.

## 1. Introduction

As IT companies provide more complex business services, meeting constant availability and expected performance levels of the enterprise applications become increasingly challenging. If the service is unavailable or unbearably slow, then users may stop using the service, which leads to a significant financial loss. For example, Amazon discovers that every 100 ms of latency costs a 1% decrease in sales, and Google reports that the traffic drops by 20% due to a 500 ms delay in response time [1]. Thus, guaranteeing both performance and availability of services is essential for a successful business.

A previous survey [1] describes the performance anomalies as an unexpected behavior with associated system failures. Due to the unpredictable and destructive nature of the performance anomalies, a system administrator must continuously inspect various system performance indicators (SPIs) that summarize the overall status of the services, such as latency or traffic. In practice, since enterprise services have hundreds of performance metrics (PMs) that have an influence on the SPI, manually diagnosing the behavior of services through SPI is time-consuming and labor-intensive. Accordingly, many large companies have built their own anomaly detection frameworks, which automatically monitor the SPI and alert the administrator when detecting abnormal behaviors in the SPIs. For instance, Microsoft [2] developed an anomaly detection algorithm to help customers track their business metrics from Bing, Office, and Azure. Yahoo [3] uses a collection of anomaly detectors to monitor and raise alerts on its various services.

Many existing anomaly detectors aim to monitor the time series of a single SPI. In other words, when the targeted SPI changes, additional steps of either designing a new anomaly detection algorithm or tuning the parameters are required. Additionally, the existing anomaly detectors seldom analyze the root causes of the anomaly, thereby failing to reduce the overhead of anomaly management and diagnosis. Although several methods to identify the causes of performance anomalies have been reported to date, they suffer from performance degradation due to the large search spaces of the PMs. For example, Peiris et al. [4] adopted the Pearson and Spearman correlation analysis that evaluates linear relationships between two variables. However, since a multitier Web system contains hundreds of PMs that affect a target SPI, the administrator should investigate all the possible pairwise combinations.

We believe that anomaly detection and root cause analysis must be performed together to provide valuable insights to help the administrators troubleshoot the performance anomalies. In light of this, a top-down approach, which identifies the performance anomalies first and then searches for the correlation between the PMs, faces several challenges. First, to launch the root cause analysis, the failure cases should be identified, implying that the performance anomalies and the corresponding PMs need to be known in advance. Second, today’s business services are hosted on a complex multitier architecture, where the tiers are physically separated and executed on separate machines. Because of such a large-scale infrastructure, the problem is often unknown, and tracing the PMs in real time is nearly impossible.

Evidently, determining the PMs relevant to the performance anomalies is a considerable challenge for the administrator. Therefore, the performance anomaly detectors should offer a good starting point to analyze the performance anomalies of the target SPI. However, efficiently selecting the most relevant PMs is challenging because of their huge search spaces. In this paper, we propose advanced causative metric analysis (ACMA), which first selects the causative metrics (CMs), among all the identified PMs, that strongly influence the variance of the target SPI. Then, the ACMA automatically performs anomaly detection and CM analysis in real time, providing helpful guidance to the administrators and minimizing the time required to analyze the behavior of the business services running on a multitier architecture. Specifically, the ACMA extracts the primary CM candidates from a set of PMs and sorts them by their impact on the variance of the target SPI. The ACMA framework offers these primary CM candidates within a few seconds when detecting the target SPI anomalies and alerts the service administrator, allowing them to handle the problem in real time. To further enhance the detection reliability in the case when the actual root causes are not in the primary CM candidates, the ACMA draws alternative CM candidates within minutes after reporting the primary CM candidates.

To demonstrate the capability of the ACMA, we evaluated its performance by applying it to a real-world enterprise service used by about 160,000 employees worldwide hosted by Samsung. The servers in the service are composed of application, operating system (OS), database (DB), and network (NW) domains. It is a mission-critical web system used by employees, so it is suitable for evaluating the performance of ACMA. It collects approximately 62,000,000 observations of the target SPI and PMs per day (approximately 8800 MB). We set the response time of the authentication service as the target SPI in the Samsung Virtual Private Network (SVPN), which consists of 28 servers (12 servers for web, 12 servers for application, and four servers for DB) and 24 network devices. The ACMA monitored the response time of the authentication service over two months (1 June 2022 to 31 July 2022). During the experiment, the ACMA detected six performance anomaly cases, where five cases were actual performance anomalies. The administrators could determine that the root causes of four of these five cases were included in the primary CM candidates, and the remaining case was located in alternative CM candidates. Therefore, we can conclude that the ACMA can maintain service availability by helping the administrator to handle the problem immediately.

Specifically, we make the following contributions to achieve our goals:

1. We identified 62 *vital* PMs that contributed the most to the target SPI in a multitier enterprise infrastructure environment and provided their corresponding threshold values.
2. Here, we present methods to extract the primary and alternative CM candidates from the *vital* PMs. Since navigating all the PMs is time-consuming, reducing the search space is necessary to derive the CMs related to the target SPI performance anomalies rapidly.
3. We used several statistical methodologies to determine whether a performance anomaly is related to the primary or alternative CM candidates, thereby bridging the gap between anomaly detection and root cause analysis. Notably, most of the existing methods adopt a top-down approach to detect outliers in the target SPI first and subsequently launch a root cause analysis, which involves multiple iterations of validation against a large amount of PMs.
4. We evaluated the effectiveness of our approach by using enterprise services. The experimental results demonstrated that the ACMA can provide the CM candidates immediately, thereby aiding the service administrator in identifying the potential causes. Based on the results obtained by implementing the ACMA to enterprise services, we conclude that the ACMA can provide deeper insights into the performance anomalies and enhance service reliability.

The rest of this paper is organized as follows. We first present the preliminaries and problem definition in Section 2. In Section 3, we describe the proposed approach and provide an overview of the ACMA framework. Next, we describe the components of the ACMA framework in Section 4 and present our evaluation results and in Section 5. In Section 6, we discuss related works, and the major conclusions drawn from the study results are presented in Section 7.

## 2. Preliminaries

### 2.1. PMs and Target SPI

In this paper, we define PM as a system feature that represents the partial state of a multitier Web system. Typically, a multitier Web system consists of four domains—application, operating system (OS), DB, and network. PMs represent the tracked detailed status of resources in each domain. For example, the OS domain includes PMs such as hostname consistency check or percentage of available free space on a logical disk. Furthermore, CPU utilization or memory utilization of switches can be a PM in the network domain. The SPI is a summarized metric that indicates the overall condition of a multitier Web system and directly represents the service availability. As mentioned in Section 1, we can set the response time as a target SPI for the search engines. Then, an anomaly detector continuously monitors the response time (i.e., target SPI) to maintain the reliability and availability of the search engine. In this section, we describe the PMs used in this study (further details are provided in Section 3.2).

Among the widely used 191 PMs, we derive 62 *vital* PMs via exploratory data analysis (EDA). Next, we select the top-*k* primary causative metric candidates (CMCs) closely related to the target SPI using the random forest algorithm. When detecting the target SPI anomaly, the primary CMs are derived using both the ACMA threshold and results of the ACMA *t*-test performed on the primary CMCs. Random forest is one of the ensemble learning methods which combines the output of multiple decision trees to reach the final result. When training an ensemble model, injecting randomness for underlying classifiers is critical for preventing the model from making biased results. The random forest algorithm achieves the classifier diversity by using the bagging technique. In the bagging method, a random sample of data in a training set is selected with a replacement to train each decision tree. Furthermore, the random forest algorithm utilizes feature bagging that chooses random features among all features to ensure a low correlation among decision trees.

**Figure 1:**
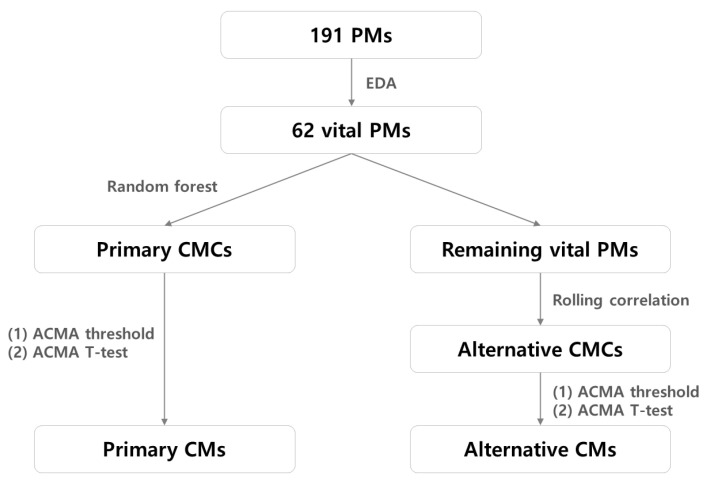
Relationship between the PMs.

In addition, we search for alternative CMs against the case that the root causes of the target SPI anomaly are not in the primary CMs. For the remaining vital PMs, excluding the primary CMCs, we analyze the relationship between the remaining vital PMs and the target SPI using rolling correlation and derive alternative CMCs. Then, we derive the alternative CMs using the same approach (i.e., ACMA threshold and *t*-test) applied for deriving the primary CMs. The details on determining the primary and alternative CMs are outlined in Section 3 and Section 4, and the relationship between the PMs is illustrated in Figure 1.

### 2.2. CMCs and CMs of Performance Anomaly

When a performance anomaly of the target SPI appears, its prompt detection and treatment are crucial to enhance the reliability and availability of the services. In this study, we defined the suspected causes of an anomaly derived by the ACMA as the “CMs” of the performance anomaly. The ACMA extracts the CMCs by first filtering out the PMs that are most relevant to the target SPI, among all the PMs. Subsequently, the ACMA determines the *primary* and *alternative* CMs via two approaches. The primary CMs are the PMs that the ACMA assumes as the actual causes of the performance anomaly and are derived using a bottom-up approach. Next, a top-down approach is applied to detect alternative CMs among the PMs, excluding the primary CMCs.

Note that collecting the abnormal behavioral data of the target SPI is nontrivial, because the anomaly cases are rare, and labeling such data one-by-one requires human intervention. Since securing a sufficient number of labeled abnormal examples for detecting CMs is challenging, we instead investigate the relationship between the target SPI and the PMs in advance based on the behavioral data when the service is in a normal state. Specifically, we rank the relevant PMs according to their significance to the target SPI; here, significance indicates the impact on the variance of the target SPI. When a target SPI anomaly occurs, the ACMA determines the primary CMs based on the relevance of the PMs via performance metric monitoring (PMM) and performance metric analysis (PMA).

**Figure 2:**
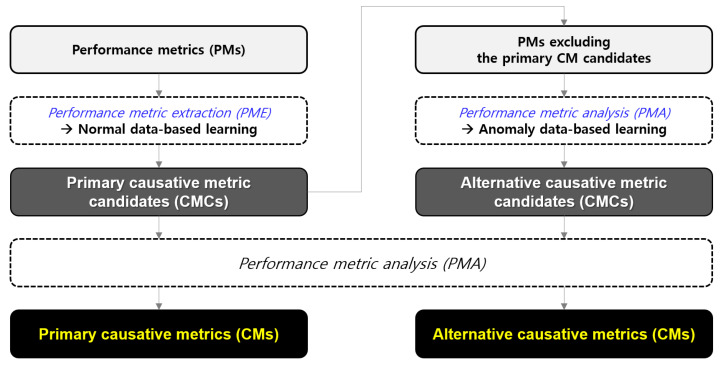
Overview of the CMCs and CMs.

While reporting the primary CMs to the administrator, a sufficient amount of abnormal behavioral data is accumulated to further analyze the vital PMs, excluding the primary CMCs. Thus, the ACMA can now launch supplementary analysis using this abnormal behavioral data to derive alternative CMs. Once the administrator receives both the primary and alternative CMs, they can leverage them as a starting point to resolve the issue promptly. The entire process is outlined in Figure 2 and elucidated in Section 4.

### 2.3. Problem Definition

**Figure 3:**
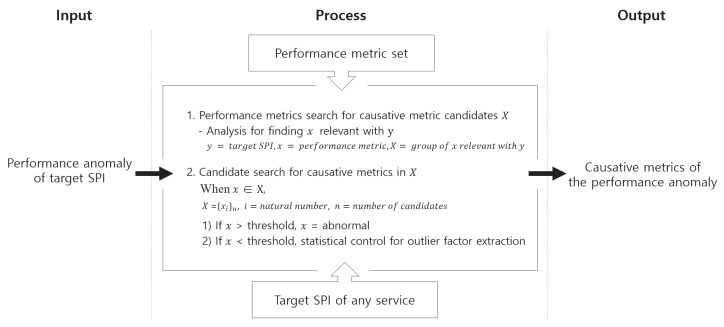
Problem definition.

Our goal is to design a framework that can correctly identify the CMs of the performance anomalies in a multitier environment, as illustrated in Figure 3. We assume that the measured values of both the target SPI and PMs are collected in a time-series form. Based on the time-series data of both the target SPI (i.e., *y* range) and PMs (i.e., *x* domain), a regression model that outputs the predicted value of the target SPI using the PMs as the input is constructed. Specifically, the regression model uses a subset of the PMs as the input of the regression model. We use the regression model to derive the CMCs relevant to the target SPI, i.e., $X={\left\{x_{i}\right\}}_{n}$; here, n represents the number of CMCs.

## 3. Proposed Method: ACMA

### 3.1. Motivating Example

This section describes how the naive approach identifies the CMs of a performance anomaly, and we compare it with our proposed method. The naive top-down approach has to search all the (CMC, target SPI) pairs to identify the CMs when a performance anomaly occurs. However, this approach of CM identification is time-consuming and thus inefficient, as evidenced by the average time for finding the CMs as well as the size of the search space listed in Table 1.

Here, enterprise network system (ENS) 1 is SVPN, and ENS2 is Samsung uReady. Note that the naive approach does not know the relevance of each PM with the target SPI in advance. Moreover, we assume that both primary CMCs and alternative CMCs have a set size of 10, respectively. Accordingly, when a performance anomaly occurs, the naive approach requires to check all combinations of primary/alternative CMCs. The total number of PMs for ENS1 is 191, and the search space for finding the CMs of a performance anomaly is ${}_{191}C_{20}$. ENS2 has a total of 186 PMs, and thus the search space is ${}_{186}C_{20}$. Thus, the naive approach needs a minimum of days to identify the CMs. This experimental study motivates us to improve the search time for finding the CMs of a performance anomaly. In addition, a general framework that can be applied in any multitier Web system with different target SPIs or PMs should be designed.

Naive approaches are vulnerable to changes in the target SPI because their prediction models should also be adjusted accordingly. To overcome this problem, we use the following strategy. First, we model a linear regression function, y=f(x)+ϵ, where *x* and *y* represent the measured values of the PMs and target SPI, respectively. This function describes the relationship between *x* and *y* without relying on specific target SPI and PMs. To build the regression function, we extract the subset of the PMs that are closely relevant to the target SPI from all the PMs in a multitier Web system. Only these PMs can be the “possible CMCs of a performance anomaly.”

In this study, we used a random forest algorithm that uses an ensemble of decision trees to derive the regression function. As mentioned before, acquiring a time-series dataset when an actual anomaly occurs is challenging. However, since we can observe the variation in *y* (i.e., the target SPI) due to *x* (i.e., PMs) in the normal state, we can use the time series in the normal state to analyze *y* according to the changes in *x*. Thus, we can use this regression relationship to assess the PMs that strongly influence the target SPI. Then, the ACMA can determine the CMs from the possible *x* candidates by comparing their threshold with the value obtained within a few seconds after the anomaly occurs; the resulting PMs are the primary CMs.

The primary CMs may not be the root causes of the performance anomaly. Thus, the ACMA follows a backup procedure, in which the alternative CMs are derived after the primary CMs are reported. Notably, the primary and alternative CMs are disjoint sets. Specifically, the ACMA analyzes the variations in the target SPI and the set of PMs, excluding the primary CMCs, using the time-series dataset after the anomaly occurrence. Similarly, any PM exceeding the threshold or deviating from the normal state is considered as an alternative CM, which are provided by the ACMA within a few minutes after it reports the primary CMs.

### 3.2. Health Check of the Target SPI

Business service in an enterprise environment that operates on various machines, such as servers, switches, and routers. Because of this heterogeneity and complexity of the multitier architecture, the detection and diagnosis of performance anomalies becomes a nontrivial and highly challenging task, even though we track each machine thoroughly. While outright system failures or crashes can be easily resolved by investigating the problematic server, the root causes of the performance anomalies are not revealed by assessing the outside behavior. Instead, we can implicitly assume that the service suffers from performance problems by closely analyzing the system logs, which contain the measurements of the PMs that track different aspects of the performance of the system.

To diagnose the performance anomalies in a multitier architecture, the ACMA sets a critical signal in the business service as a target SPI and performs *health check* (HC) to assess its normality. HC represents the process of identifying any performance anomaly of the target SPI. This process also includes extraction of the CMs relevant to the performance anomalies. In summary, HC consists of two procedures: monitoring the target SPI and diagnosing the CMs of the performance anomaly.

In this study, we set the response time (latency) of the user’s request (e.g., Web page or Web URL) as the target SPI. To narrow the search space of the PMs, we only deal with a total of 191 PMs provided by the major vendors of each equipment: 41 from the OS domain, 63 from the DB domain, 17 from the storage domain, 45 from the network domain, and 25 from the application domain. Moreover, the initial threshold for each PM is determined through the domain knowledge of the enterprise system. For example, we exclude the Check_storage_path from PM owing to its low impact on the response time. In brief, we extract 62 vital PMs among the 191 PMs and describe them in detail in the following section.

### 3.3. PMs of the ACMA

In this section, we list the vital PMs and their threshold values. We first conducted EDA on the total of 191 PMs provided by well-known vendors, such as HP, Dell, Cisco, F5 Networks, and Jennifer [5]. In detail, we used the PM measurement data of past two months for SVNP [6] and Samsung uReady [7] services and derived a total of 62 vital PMs. We also set the threshold value of each vital PM based on the initial recommended values provided by the vendors or those based on the analysis of the monitoring data of past two months.

**Exploratory Data Analysis (EDA)**. Among the 191 PMs, some are relatively less relevant or unrelated to the target SPI. We first screen out the PMs associated with the target SPI using Caling’s method [8]. Specifically, we set the criteria for determining whether a PM can be a vital PM based on the median (M), quartile (Q), and interquartile range (IQR). These M, Q, and IQR values of the PMs are derived from the time series data during a specific period. If the PM measurement at one point is outside the range [M-multiplier*IQR, M+multiplier*IQR], then it is excluded from the set of vital PMs. Here, the value of IQR is Q3 (median of upper half)-Q1 (median of lower half), and the default multiplier of Carling is 2.3.

The monitoring frequency of individual PM is set as not to affect the overall detection performance of the ACMA. PMs that can have a critical impact on the system need high-frequency monitoring, for instance, every second. However, low-monitoring frequency is acceptable for PMs with a low system impact. For example, higher CPU usage directly affects the service delivery capability of the server. Accordingly, *Process_CPU_Usage*, which measures the CPU utilization of the server, is monitored every second. On the other hand, *TableSpaceFreeSpace*, which measures the remaining space of DB, does not immediately affect the system reliability and is monitored every 60 min.

**Figure 4:**
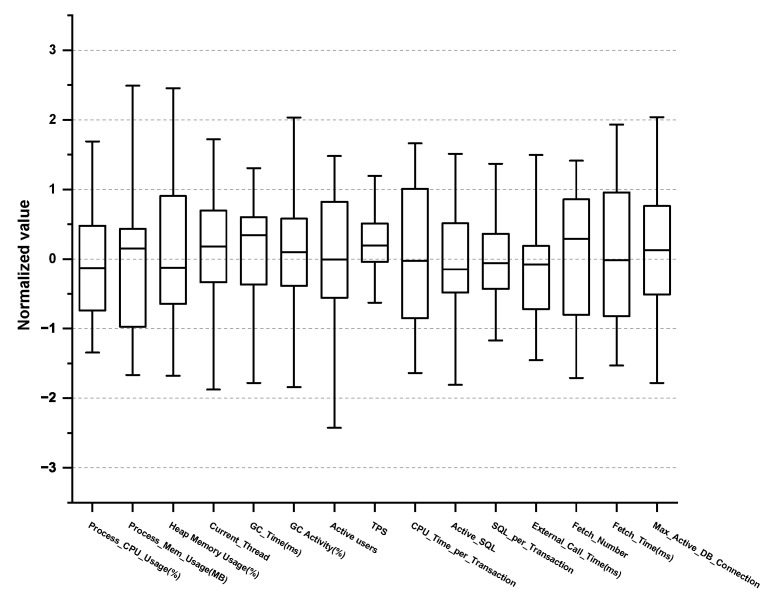
Box plot of vital PMs in the application domain.

**Figure 5:**
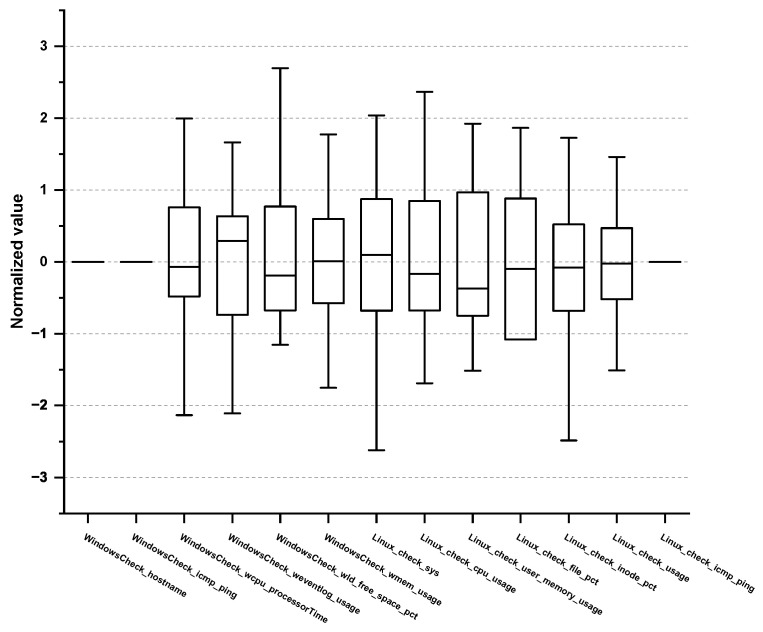
Box plot of vital PMs in the OS domain.

Furthermore, we examined the max/min, mean, variance, and quartile of each PM in the normal/abnormal behavioral data to eliminate any PMs that do not follow the normal distribution. For example, check_cpu_util, which measures the CPU usage, follows normal distribution, and thus, we selected it as a vital PM, whereas cron, which measures the cron log or database batch program, does not have a direct relationship with the target SPI, and thus we excluded it. Subsequently, we consulted the expert group and excluded any PMs that do not require monitoring. Consequently, a total of 62 vital PMs were selected, as shown in Table 2, Table 3, Table 4 and Table 5. Concretely, 15 PMs were derived from the application domain and 13 PMs from the OS domain as outlined in Table 2 and Table 3, respectively. Box plots for 15 PMs (Application) and 13 PMs (OS) are shown in Figure 4 and Figure 5.

**Figure 6:**
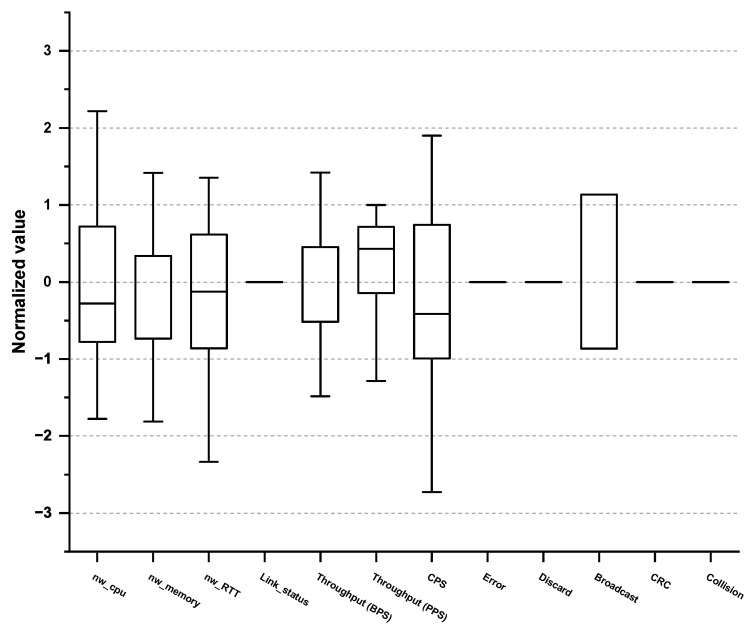
Box plot of vital PMs in the network domain.

**Figure 7:**
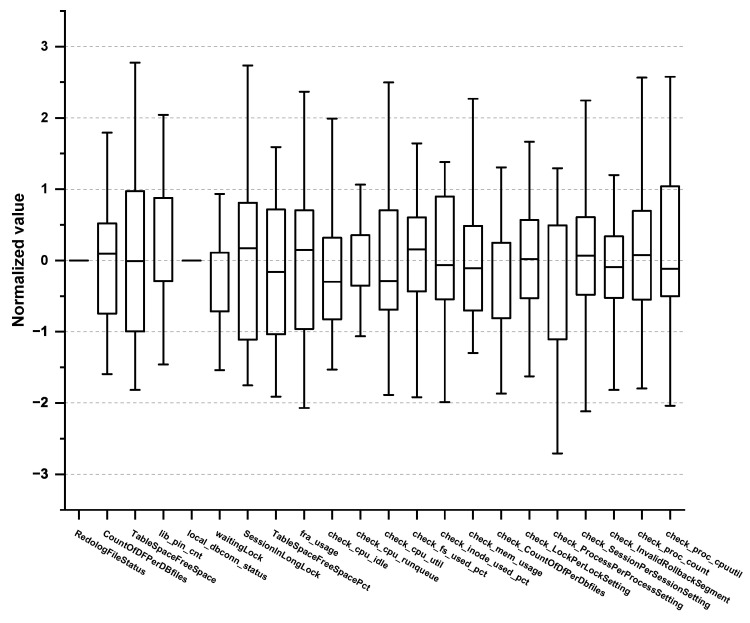
Box plot of vital PMs in the DB domain.

Moreover, 12 PMs originate from the network domain and 22 PMs from the DB domain as listed in Table 4 and Table 5, respectively. Box plots for 12 PMs (Network) and 22 PMs (DB) are shown in Figure 6 and Figure 7. To the best of our knowledge, no paper reports such organized PM tables. Most studies attempt to find the root causes of the performance anomaly in a top-down manner based on every combination of the possible causes. This approach requires a significant amount of time and resources to figure out the root cause of the performance anomalies. However, the ACMA can exploit these predefined PMs obtained from the EDA, leading to a quicker and more accurate detection of the actual root causes.

The average coverage of the vital 62 PMs in the ACMA is 82.9%. Specifically, according to the web application market study conducted by Gartner [9], the usages of various vendors used by global companies, such as Google or Samsung, are listed as follows: Apache (41.7%), Nginx (26.4%), Microsoft Internet Information Services (IIS) (12.5%), LiteSpeed (2.2%), and others (17.2%). Since ACMA supports Apache, Nginx, and IIS, it can cover 80.6% of the application domain. According to Gartner [10], the server market share is 56.8% for Windows server, 21.3% for Linux server, 5.7% for IBM AIX, 4.1% for HP-UX, and 12.1% for others. The ACMA supports the Windows and Linux servers, covering 78.1% of the OS domain.

The ACMA uses a simple network management protocol (SNMP) to monitor the vital PMs in the network domain. Since all the global vendors, such as Cisco, F5, and Jennifer support the SNMP [11,12,13,14,15], the ACMA is capable of covering every vital PM in the network domain. Last, according to the study on the DBMS domain by Gartner [16], vendor products and their usages are as follows: Oracle (31.1%), Microsoft DB (24.8%), Amazon Dynamo DB (13.5%), IBM DM2 (10.4%), SAP (6.9%), and others (13.3%). Since the ACMA supports Oracle, Microsoft DB, IBM DB2, and SAP, it can cover 73.2% of the DBMS domain. In summary, the average coverage of all the four domains (Applications, OS, Network, and DB) is 82.9%. Notably, the PMs used in the ACMA do not cover any multitier system, although they are effective in most enterprise systems. Further, the ACMA still operates even if we manually add other PMs. Thus, we can conclude that the ACMA can be adapted to any enterprise environment.

### 3.4. Overview of the ACMA

**Figure 8:**
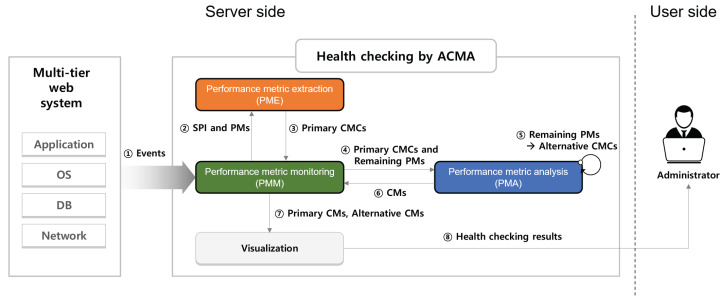
Overview of performance anomaly detection and identification of CMCs and CMs by the ACMA framework.

The architectural overview of the ACMA is illustrated in Figure 8. The ACMA consists of four components: *performance metric extraction* (PME), *performance metric analysis* (PMA), *performance metric monitoring* (PMM), and *visualization*. We will describe each component of ACMA in detail in Section 4.

Recall that HC represents the process of detecting the performance anomalies and deriving their CMs. Deriving the CM consists of two steps: (1) finding the primary CMCs and CMs and (2) identifying alternative CMCs and CMs. Here, we clarify each step as follows:

**Finding primary CMCs and CMs**: When the target SPI shows a normal behavior (i.e., the services operate as usual), the PMM transmits the measurements of the vital 62 PMs to the PME. Then, the PME builds the regression model using a random forest algorithm based on the vital 62 PMs and determines the primary CMCs. Next, the vital 62 PMs, including the primary CMCs, are tracked through PMM. If the target SPI shows an anomalous behavior (i.e., deviating from the ACMA threshold), then the PMM concludes that the performance anomaly has occurred and delivers the measurements of the target SPI and primary CMCs to the PMA. The PMA runs the *primary CM analysis* to derive the primary CMs from among the primary CMCs. Then, the PMA transmits the primary CMs back to the PMM, which sends the measurements of the primary CMs to *visualization*. Finally, *visualization* displays the values of the primary CMs to the users.

**Finding alternative CMCs and CMs**: After detecting the performance anomaly and primary CMs, the PMM transmits the measurements of the remaining vital PMs to the PMA (for example, if we choose ten primary CMCs out of 62 vital PMs, then there are 52 remaining vital PMs). Then, the alternative CMCs are extracted through an *alternative CM analysis*. The ACMA compares the measurement of the alternative CMCs with their threshold and determines them as alternative CMs if the alternative CMCs exceed their threshold. These alternative CMs are also sent to the PMM, which then passes the measurement values to *visualization*. Finally, *visualization* displays the received alternative CMs to the administrators.

**Figure 9:**
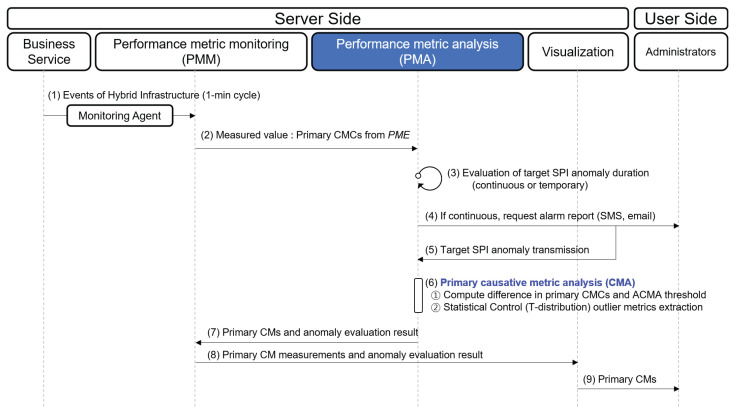
Procedure for detecting performance anomaly and finding primary CMs.

The procedure for reporting the primary CMs is depicted in Figure 9. Three components are involved in finding the primary CMs: PMM, PMA, and visualization. (1) Event logs that hold the measurement values of the PMs are collected every minute from a cloud or on-premise infrastructure, which consists of monitoring agents and communication protocols, such as Filebeat [17], Jennifer [5], Zabbix [18], and SNMP. (2) PMM checks the current status of the primary CMCs derived from the PME (see Section 4.1). (3) PMM runs *false alarm reduction* to find continuous performance anomalies. Since the value of target SPI fluctuates owing to various reasons, there can be numerous points that exceed the threshold. Thus, the ACMA only handles performance anomalies that the observed value of the target SPI consistently exceeds its threshold. (4) If an anomaly is determined as a continuous one, ACMA sends alarms to the administrator. (5) While forwarding alarm signals, PMA receives the continuous anomaly and starts to analyze its primary CMs. (6) If any of the primary CMCs exceed their threshold values, the corresponding primary CMCs are determined as the primary CMs. Even though the measured values of the primary CMCs do not exceed their threshold, the PMA further analyzes the *t*-distribution of the primary CMCs to examine whether they exhibit unusual trends. (7) PMA sends the primary CMs to the PMM. (8) PMM sends the measurement values of the primary CMs to visualization. (9) Visualization demonstrates which primary CMs strongly influence the performance anomaly through the dashboard.

**Figure 10:**
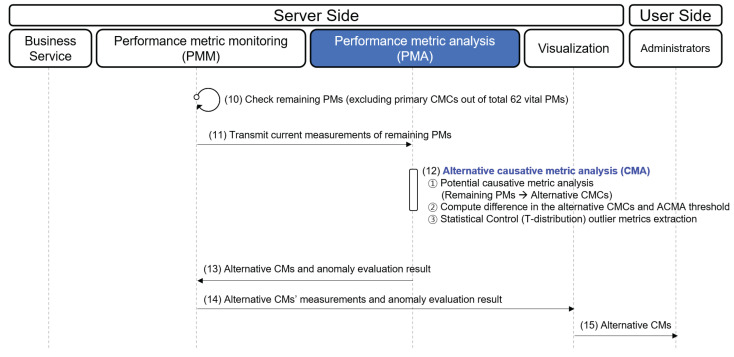
Procedure for finding alternative CMs.

Next, we illustrate the procedure for reporting the alternative CMs in Figure 10. The ACMA begins these steps after completing step 9 in Figure 9. (10) PMM checks the current status of the remaining vital PMs (excluding primary CMCs from all the vital PMs). (11) PMM delivers the current measurements of the remaining vital PMs to the PMA. (12) Alternative CMCs are derived through an *alternative CMs analysis*. Then, the PMA considers any alternative CMC greater than its threshold or that showing outlier behavior based on *t*-distribution as an alternative CM. (13) The alternative CMs are sent to the PMM. (14) The measurements of alternative CMs and their *t*-distribution are delivered to visualization. (15) Visualization highlights the alternative CMs that exceed their threshold value through the dashboard.

## 4. Description of Acma Components

**Figure 11:**
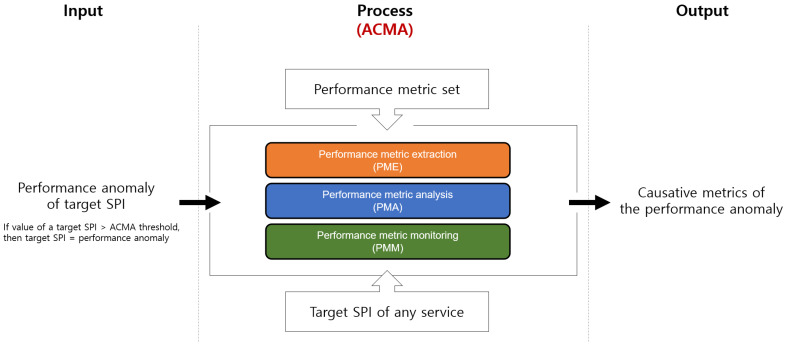
ACMA model for detecting the performance anomaly.

In this section, we outline each component of the ACMA in detail as illustrated in Figure 11.

### 4.1. Performance Metric Extraction (PME)

**Figure 12:**
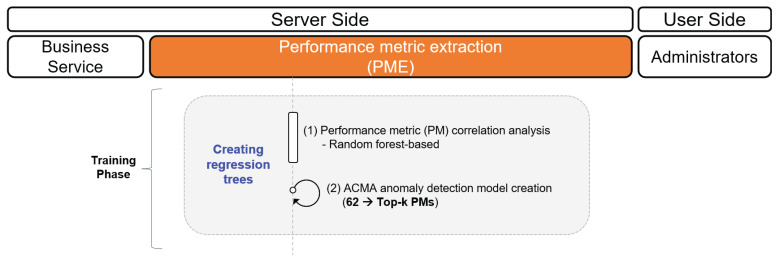
Procedure for creating the ACMA model.

The main objective of the PME is to compute the primary CMCs out of the vital 62 PMs closely related to the target SPI, as shown in Figure 12. Specifically, the PME performs the following steps: (1) PME builds a random forest model with the collected time-series data of the vital 62 PMs. (2) PME evaluates the impact of PMs on the variance of the target SPI via the trained random forest model and chooses the top-*k* PMs among them.

Specifically, we implement a general regression model to adapt any target SPI and PMs. Recall that this model computes a prediction value of the target SPI (i.e., y=f(x)+ϵ). The random forest builds this regression function that represents the relationship between *x* (i.e., PMs) and *y* (i.e., target SPI). Through this regression model, we can rank the PMs according to their influence on the variance of the target SPI. Then, the ACMA tracks the behavior of the top-*k* PMs to detect the performance anomalies through a bottom-up strategy. We set the value of *k* as 10 in our case. Note that the value of *k* can be varied depending on the administrator’s settings. Next, we describe the process of building the random forest model.

**Performance metric correlation analysis**. The PME uses an ensemble of decision trees [19,20,21], known as a random forest [22,23,24,25,26]. Specifically, the random forest leverages the power of multiple decision trees generated from different subsets of the original data. When determining the final prediction value of the target SPI, random forest follows the majority rules (classification) or computes the average value (regression). In our setting, *y* represents the measured value of the target SPI, ($x_{1},x_{2},\ldots ,x_{m}$) represents the measured values of the PMs, and *B* represents the number of decision trees. Finally, T(x) represents the prediction value calculated from a single tree *T*.

**Figure 13:**
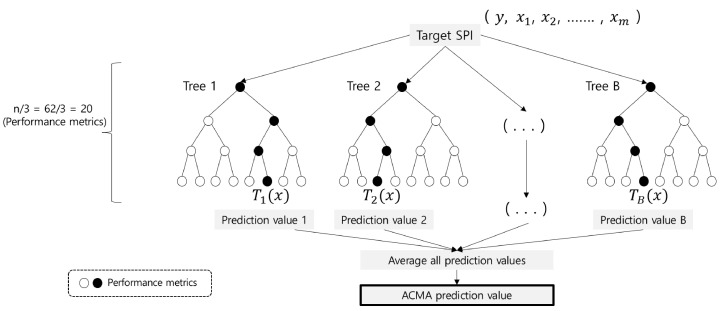
Concept of the ACMA regression model.

After all the decision trees are trained, the final prediction value of the target SPI is estimated by averaging the prediction value T(x) from each decision tree, where the prediction value is $\hat{f}\left(x\right)=\frac{1}{B}\sum_{i=1}^{B}T_{i}\left(x\right)$. From this value, we can model the regression function $\hat{f}\left(x\right)+\epsilon$, which calculates the most similar prediction value of the target SPI by minimizing the error ϵ. By comparing $\hat{f}\left(x\right)+\epsilon$ with the current measured value of the target SPI *y*, we can determine whether the system suffers from performance anomalies. Once the performance anomaly occurs, the ACMA examines the abnormality of the most relevant PMs and provides them within several seconds. In Figure 13, we describe the three steps used to train the ACMA random forest model in detail.

**Step 1**. Generate *B* bootstrap samples, L${}_{1}$, …, L${}_{B}$, from the training dataset consisting of the observed value of PMs, where L${}_{b}$ represents the extracted samples with repeats allowed. By using different bootstrap samples, we can build multiple decision trees from a single training dataset. Recall that the bootstrap sample is used for bagging (bootstrap aggregating) technique, where each decision tree in the random forest is trained by random training set with replacement.

**Step 2**. In regression function modeling, our aim is to determine the most influential predictors (i.e., the subset of vital PMs) that should be included in the primary CMCs. We can identify the best vital PMs based on statistical characteristics, such as importance or accuracy. Specifically, we grow a single random forest tree T${}_{b}$ using a random set of vital PMs. For each bootstrapped data L${}_{b}$, we randomly select *m*vital PMs; a general rule of thumb is m=n/3, where *n* is the total number of the vital PMs [27]. Note that the criteria for choosing the best vital PM to split the tree node depend on the data type of the target SPI. When the target SPI has categorical values, we use Gini Impurity and mean decrease accuracy. Conversely, we use mean squared error (MSE) for the numerical target SPI. Since the target SPI in our case is latency (i.e., numerical value), we select the MSE for evaluating the importance of the vital PMs.

**Step 3**. Finally, we combine the ensemble of trees {T${}_{b}$}${}_{1}^{B}$, where we estimate the final prediction value of the target SPI by averaging the individual bootstrap predictions, as shown in Equation (1).

(1) $${\hat{f}}_{rf}^{B}\left(x\right)=\frac{1}{B}\sum_{b=1}^{B}T_{b}\left(x\right)$$

Equation (2) shows the MSE between the observed value (*y*) and the predicted value ($\hat{f}$) of the target SPI. By closely looking at the variation of MSE according to the different sets of PMs, we can determine the relevance between the vital PMs and the target SPI. Specifically, the greater the rate of changes in MSE increases, the more influential a vital PM is to the target SPI. MSE between the observed value (*y*) and the predicted value ($\hat{f}$) can be calculated as in Equation (2).

(2) $$MSE_{PME}=\frac{\sum_{i=1}^{B}{\left(y-{\hat{f}}_{i}\left(x\right)\right)}^{2}}{B}$$

Generally, we can obtain a better prediction model if we increase the number of bootstraps. However, determining the optimal number of bootstraps is challenging, because at some point, more bootstraps will merely increase the computation time without improving the prediction accuracy. Thus, we should consider the trade-off between computation time and model accuracy. Since we sample the bootstrapped data with repetition, some measurements of the vital PMs are not included in any bootstrap sample, and these measurements are called out-of-bag (OOB). We can calculate the OOB error by using OOB as the validation sample for evaluating the regression accuracy. The OOB error stops decreasing when approximately 1073 trees are generated in our experiments. Accordingly, we set the number of bootstrap samples as 1073.

### 4.2. Performance Metric Analysis (PMA)

**Figure 14:**
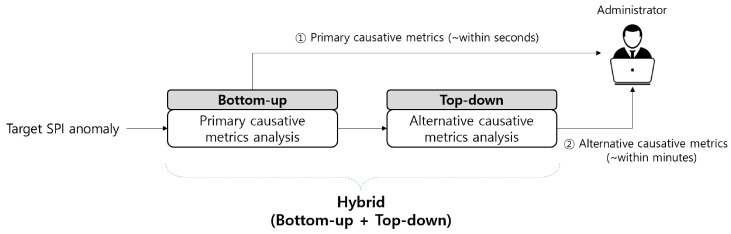
Process of finding CMs in PMA.

PMA mainly performs the hybrid process depicted in Figure 14, which involves the following two steps: (1) PMA conducts a *primary CM analysis* to determine the primary CMs among the primary CMCs derived by the PME (bottom-up). (2) After reporting the primary CMs, the PMA runs an *alternative CM analysis* that derives both the alternative CMCs and alternative CMs among the remaining vital PMs (top-down).

**Figure 15:**
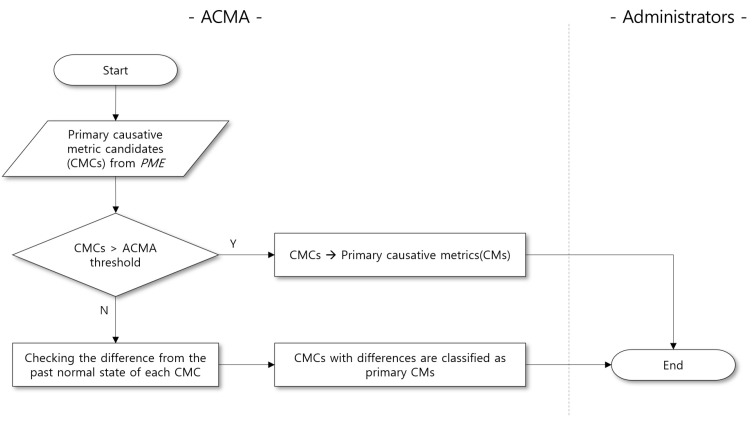
Flowchart for determining primary CMs.

We define a rule to determine whether a particular primary CMC can be the primary CM in Figure 15. Specifically, the PMA first checks the differences between the measured values of the primary CMCs and their thresholds. Although the measured values of the primary CMC do not exceed the threshold, the PMA further checks the *t*-distribution of each primary CMC if it deviates significantly from that of the normal status.

First, we briefly describe the techniques of applying *t*-distribution to extract the primary CMs. It is challenging to obtain sufficient time-series measurements at actual anomaly occurrences. Thus, our objective is to learn the patterns of the target SPI when the service is in a normal state instead. The values of the target SPI and its relevant PMs fluctuate even if they are in the normal state. Hence, the target SPI and PMs have variations in the “safe region” where they do not exceed the ACMA threshold when the system shows normal behavior. PMA attempts to learn the relationship between the target SPI and its relevant PMs under the safe region. Then, PMA derives the primary CMs by measuring how far an observation is from this safe regions even though the primary CM does not exceed the threshold.

**Primary CM analysis**. PMA uses a *t*-test to compare the *t*-distribution of each primary CMC. The *t*-test is a method of comparing two sample groups that contain observations drawn from the population [28,29,30]. Note that we assume the measurements of the target SPI and PMs follow the normal distribution. We aim to test whether the means of observation when the service in normal state and anomaly cases are significantly different. One problem with this approach is that we do not know the population variances in advance. To overcome this problem, we use *t*-distribution instead of normal distribution.

The *t*-distribution differs from the normal distribution, because it allows comparison between sample groups when the variance of the population is unknown. In general, the *t*-distribution becomes similar to the normal distribution for 30 or more degrees of freedom [31].

Then, let us assume *a null hypothesis* $H_{0}$ indicating that the observed values μ in the normal state and the incoming observations *x* are drawn from the same distribution. To falsify the null hypothesis (i.e., *x* comes from an abnormal state), we first choose the *significance level*. The significance level refers to the probability of rejecting the null hypothesis, and the commonly used significance levels α are 0.01 or 0.05. With this predetermined significance level and the degrees of freedom, we can compute the *t-critical value* that gauges the confidence interval.

Second, we outline the process to determine alternative CMs. Note that we cannot leverage labeled data to identify primary CMs. As a result, actual causes of the performance anomaly might not exist in the primary CMs. However, while reporting the primary CMs, sufficient accumulated observations at the time of anomaly occurrence are now available, and we can take advantage of it. In other words, we can double-check the relationship between the target SPI and the remaining vital PMs.

**Figure 16:**
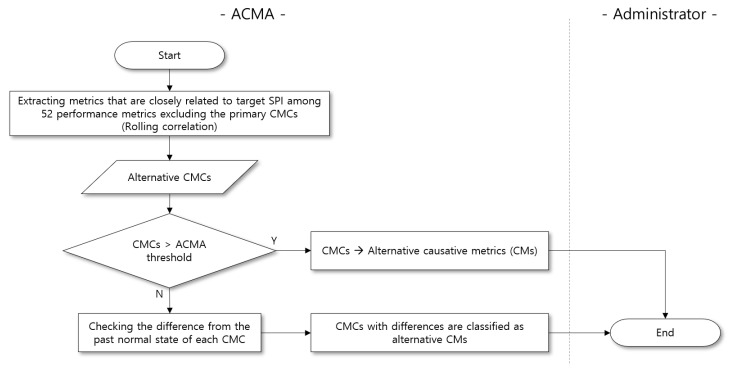
Flowchart diagram for finding alternative CMs by ACMA.

The PMA continuously monitors the variation of the remaining vital PMs and the target SPI at the time of anomaly occurrence. Then, PMA derives the alternative CMCs from the remaining vital PMs through the *alternative CM analysis*. Similar to the process of deriving the primary CMs, if an alternative CMC shows an abnormal behavior, such as exceeding the ACMA threshold or exhibiting a different pattern from the normal state, then we consider it as an alternative CM; the corresponding process is illustrated in Figure 16.

**Alternative CM analysis**. In this process, the PMA calculates the rolling correlation (correlation between two time series data on a rolling window) to derive alternative CMCs. Specifically, the PMA calculates the Pearson correlation [32,33], which measures the strength of association between the remaining vital PMs and the target SPI, using the latest 100 min of observations.

### 4.3. Performance Metric Monitoring (PMM)

**Figure 17:**
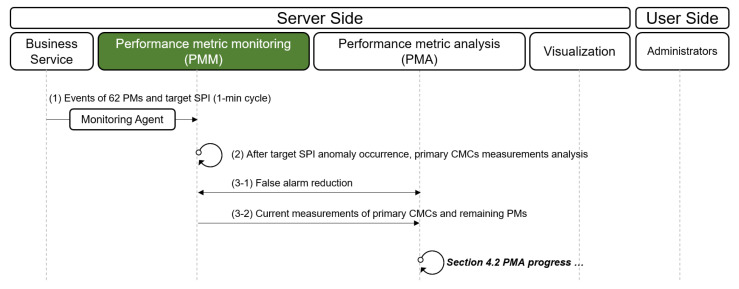
Procedure for PMM.

PMM determines whether the system suffers from performance anomaly or not. PMM searches for anomalous symptoms by comparing the incoming target SPI observations with the expected values learned from the normal pattern. This approach can identify novel and unseen anomalies, but it can suffer from a high rate of false alarms. Note that the PMM focuses on finding sustained anomalies that continuously violate the threshold, rather than point anomalies (i.e., short-duration or one-time). Since frequently reporting such point or short-duration anomalies increases the service administrator’s operational burden, the PMM conducts a false alarm reduction procedure as shown in Figure 17.

**False alarm reduction**. Once the measurements of all the vital PMs are collected, the ACMA checks whether the average value of the vital PMs during a predefined time interval exceeds the threshold. Following this procedure, the ACMA reduces the events that can be automatically resolved without requiring any additional action by the administrator, even if the observations momentarily exceed the threshold. In our implementation, we set the default time interval to three minutes.

Next, we explain the monitoring procedures. (1) The measurements of the target SPI and vital PMs are collected by default every minute. (2) When the target SPI exceeds the ACMA threshold, the PMM checks whether the measurements of the primary CMCs exceed the ACMA threshold. (3-1) The PMM performs a false alarm reduction, and (3-2) the ACMA reports the current measurements of the primary CMCs and remaining PMs to the PMA. The subsequent process is identical to that of the PMA described in Section 4.2.

### 4.4. Implementation of the ACMA Framework

**Figure 18:**
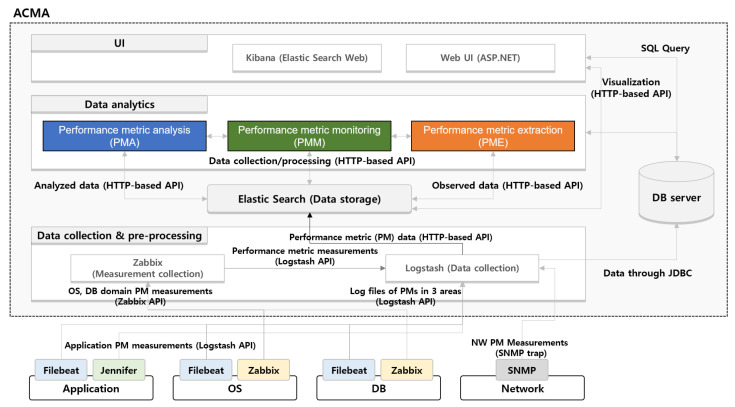
Overall system architecture of the ACMA.

In Figure 18, we briefly describe the overall system architecture of the ACMA framework, which consists of four different areas: UI, Data analytics, Elastic Search [34], and Data collection and preprocessing. When the data type of the PMs is text, the Filebeat [35] agent transfers the measurements through the Logstash API to Logstash in the Data collection and preprocessing area. When the PMs have numerical type values, the application uses the Jennifer [36] agent to forward the measurements to Logstash in the Data collection and preprocessing area. Zabbix [37] collects the PM values of the OS and DB domains and transmits them to Zabbix in the Data collection and preprocessing area. In order to manage the entire log, any information collected by the Zabbix agents is transmitted to Logstash. Last, the PM values in the network domain are transmitted to Logstash through an SNMP and SNMP Trap [38].

Real-time log and measurements collected by Logstash are further transmitted to Elastic Search using an HTTP-based API. Based on the saved measurements in Elastic Search, the *PMM, PME*, and *PMA* perform the analyses described in Section 4. The ACMA results are displayed to a service administrator through Kibana [39] in the UI area, which is a Web interface for Elastic Search.

The ACMA consists of five servers to manage and operate the monitoring and analytics functions; the ACMA web server, ACMA analytics server, ACMA search server, ACMA collection server, and ACMA DB server. The ACMA Web server is a UI server that hosts Kibana. The ACMA analytics server conducts data analysis using R and Python. The ACMA search server stores the measurement data of the target SPI and PMs and uses Elastic Search as a search engine. The ACMA collection server performs measurement collection and preprocesses the measurements collected from the *Filebeat, Jennifer, Zabbix* agents and SNMP. Subsequently, the ACMA collection server organizes the collected measurements in a consistent format and stores them into Logstash. Last, the ACMA DB server is an Oracle-based RDBMS server. Hardware information of all the five servers is outlined in Table 6.

## 5. Evaluation

This section presents case studies for performance verification of the ACMA through the SVPN, which is a Web application that allows access to the company’s intranet from public Internet. It is a mission-critical web application used by more than 160,000 employees worldwide. The SVPN consists of a multitier infrastructure, including 12 Web servers (in New Jersey, London, India, Beijing, South Korea, and Singapore), 4 DB servers, 24 network equipment, and 12 Web application servers. Approximately 62,000,000 observations of the target SPI and PMs are collected per day, with a file size of around 8800 MB. Accordingly, we believe that this setting is suitable for verifying the performance of the ACMA.

### 5.1. CMCs in Experimental Settings

**Figure 19:**
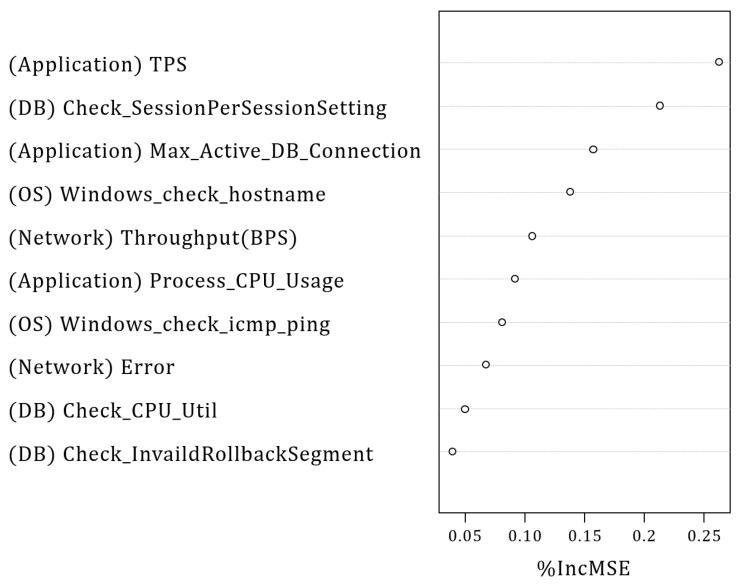
%incMSE values of Primary CMCs of the target SPI.

The two most important services of the SVPN are two-factor user login and accessing the network. The SVPN services do not allow a slow response time or timeout in the login and network access page. Thus, we set the response time of the SVPN login webpage as the target SPI. Using the latest measurement data of one month, the PME selects the top 10 primary CMCs of the target SPI according to the correlation with the target SPI using the random forest model as shown in Figure 19. Recall that the ACMA evaluates the correlation using the MSE defined in Equation (2).

We select the top 10 primary CMCs that show the highest increase rate of MSE (%incMSE) from the vital 62 PMs. The detailed %incMSE values of each primary CM are as follows: (Application) Transaction Per Second (TPS) = 0.27, (DB) Check_SessionPerSession Setting = 0.21, (Application) Max_Active_DB_Connection = 0.16, (OS) Windows_check_host name = 0.14, (Network) Throughput (BPS) = 0.11, (Application) Process_CPU_Usage = 0.09, (OS) Windows_check_icmp_ping = 0.08, (Network) error = 0.07, (DB) Check_CPU_Util = 0.05, and (DB) Check_InvaildRollbackSegment = 0.04. Based on these primary CMCs, we summarize the experimental setting in Table 7.

### 5.2. Case Studies

We monitored the SVPN for two months (1 June 2022 to 31 July 2022). A total of six target SPI performance anomalies were detected during the monitoring period, and five of them were actual performance anomalies, while the remaining one was an unknown error. Table 8 shows the detailed summary of the target SPI anomalies detected during the experiment periods. Each row of Table 8 displays the description of anomaly, root cause location, and provided CMs ranked by ACMA for each case. We further demonstrate below the primary and alternative CMs of the five actual anomaly cases and present the heatmaps that visualize the degree of correlation between the CMs and the performance anomaly.

**Figure 20:**
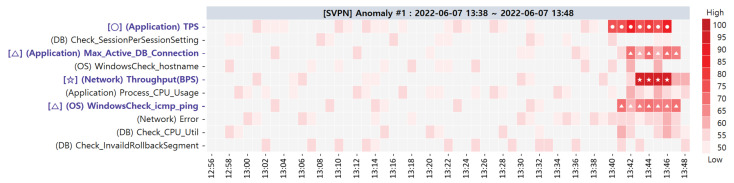
Primary CMCs/CMs of target SPI anomaly #1 via ACMA visualization.

**Target SPI Anomaly #1**. Figure 20 shows the visualization result of the ACMA provided to the service administrator at the time of anomaly #1 occurrence. The color of the heatmap becomes darker as the distances between the values of the primary CMCs and their thresholds increase. We arrange the distances along the scale of 50 to 100. In short, for a greater distance, the ACMA considers the primary CMC as the root cause. The index on the left side of Figure 20 describes the (Domain, Primary CMC) pairs. The primary CM, whose *t-statistic value* exceeds the *t-critical value* and ACMA threshold, is displayed with the symbol [✩]. The symbol [○] represents the primary CM that exceeds the ACMA threshold but does not exceed the *t-critical value*. Last, the symbol [△] indicates the primary CM that exceeds the *t-critical value* value but does not exceed the ACMA threshold.

The primary CMs likely to be the root causes are as follows: [✩] (Network) Throughput (BPS), [○] (Application) TPS, [△] (Application) Max_Active_DB_Connection, and [△] (OS) WindowsCheck_icmp_ping. (Application) TPS and (Application) Max_Active_DB_ Connection caused an overload on the IIS of the SVPN web server. As a result, the number of users who could not complete the login process increased, resulting in an increased (Network) throughput (BPS) and (OS) WindowsCheck.icmp_ping. These problems affected the SVPN response time and led to a target SPI anomaly.

The summary of the alternative CMCs was also provided to the service administrator as depicted in Table 9. In the case of anomaly #1, the root causes existed in the primary CMs. Consequently, the administrator solved this problem by rebooting the web server IIS and taking steps to make the response time normal. As a result, the measurements of all the primary CMs returned to the normal range.

**Figure 21:**
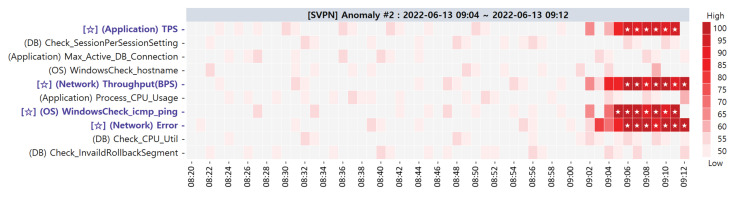
Primary CMCs/CMs of target SPI anomaly #2 via ACMA visualization.

**Target SPI Anomaly #2**. This anomaly occurred because wrong configuration PM values were inserted in the network domain. The heatmap visualization of the ACMA is illustrated in Figure 21. The primary CMs that are highly suspected to be the actual root causes are [✩] (Application) TPS, [✩] (Network) Throughput(BPS), [✩] (OS) WindowsCheck_icmp_ping, and [✩] (Network) Error. Alternative CMCs are depicted in Table 10. Similar to the case of anomaly #1, the root causes of anomaly #2 were all included in the primary CMs. After restoring the configuration value in the network equipment, the measurements of the four primary CMs returned to a normal range.

**Figure 22:**
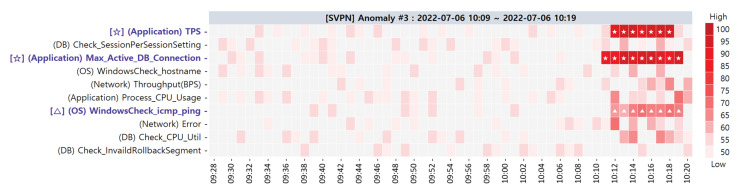
Primary CMCs/CMs of target SPI anomaly #3 via ACMA visualization.

**Target SPI Anomaly #3**. Anomaly #3 resulted from an error in the network time protocol included in the network domain PMs. The corresponding visualization result of the ACMA is shown in Figure 22. After the detection of anomaly #3, the primary CMs suspected to be the root causes are as follows: [✩] (Application) TPS, [✩] (Application) Max_Active_DB_Connection, and [△] (OS) WindowsCheck_icmp_ping.

Similarly, the ACMA prepared alternative CMCs to ensure a smooth execution in the case the root causes are not present in the primary CMs. The selected alternative CMCs are depicted in Table 11. According to the SVPN operational manual, the primary CMs (TPS, Max_Active_DB_Connection, WindowsCheck_icmp_ping) are closely related to the Internet Information Service (IIS) hang of the Web server and well-known root causes that delay the connection to the SVPN Web page. The administrator resolved this anomaly by resetting the web server IIS, and the primary CMs returned to their normal values.

**Figure 23:**
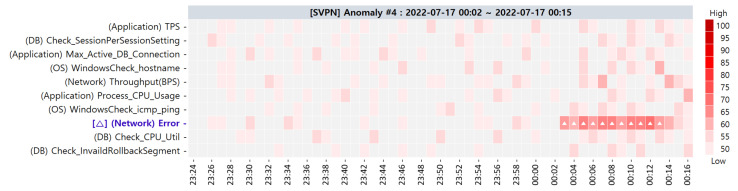
Primary CMCs of target SPI anomaly #4 via ACMA visualization.

**Target SPI Anomaly #4**. While the ACMA rapidly provided the primary CMCs/CMs of anomaly #4 to the administrator, they could not find the root causes of this anomaly among the primary CMCs/CMs. Soon after receiving the alternative CMCs/CMs, the administrator could identify the root causes that existed in the alternative CMs. The administrator could resolve target SPI anomaly #4 by carefully controlling the PMs in the alternative CMs. We first demonstrate the visualization result of the primary CMCs in Figure 23.

Anomaly #4 occurred because of an expired secure sockets layer (SSL) certificate in the SVPN Web server, which resulted in the HTTPS connection error. Accordingly, the response time of the webpage exceeded the threshold. Although we confirmed this anomaly and received the primary CMCs, we could not locate the root cause. We highly suspected [△] (Network) Error based on the primary CMs, but it was not the root cause. After receiving the alternative CMCs a few minutes later, we could determine the root causes of anomaly #4 as outlined in Table 12. The alternative CMCs, whose *t-statistic values* exceed the *t-critical values*, are highlighted in bold.

The highly suspected alternative CMs that exceeded the threshold are [✩](Network) CPS, [○](Application) Process_Mem_Usage(MB), [○](OS) WindowsCheck_wcpu_processor Time, [✩](Network) Discard, [○](Application) Current_Thread, [✩](Network) Throughput (PPS), [○](Network) Collision, and [○](Application) GC_Time(ms). According to the SVPN service troubleshooting guide, when [✩](Network) Discard, [○](Application) Current_Thread, and [○](Application) GC_Time(ms) show exceptional patterns, they are deeply correlated with problems in communication with the HTTPS protocol. The HTTPS protocol provides security to the HTTP protocol by allowing encrypted communication using an SSL certificate. Note that the SSL certificate has information about the issuer, identity of the owner, public key, and validity period. The SSL certificate guarantees a secure communication during the validity period and is not automatically renewed when its validity period expires. When the validity period of an SSL certificate expires, connection via the HTTPS protocol is not established, resulting in an anomalous behavior in these CMs. By examining these alternative CMs, the administrator concluded that the problem was due to the SSL certificate since the Web server and NW equipment were all faultless, even though the Web page could not be accessed. The service administrator issued a temporary SSL certificate to resolve this anomaly.

**Figure 24:**
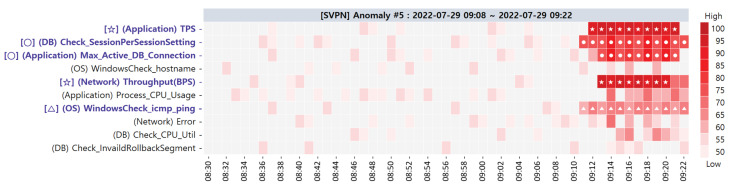
Primary CMCs/CMs of target SPI anomaly #5 via ACMA visualization.

**Target SPI Anomaly #5**. The heatmap visualization of the primary CMs of this anomaly is shown in Figure 24. After detecting anomaly #5, the primary CMs suspected to the root causes are as follows: [✩] (Application) TPS, [○] (DB) Check_SessionPerSessionSetting, [○] (Application) Max_Active_DB_Connection, [✩] (Network) Throughput(BPS), and [△] (OS) WindowsCheck_icmp_ping; they were immediately reported to the service administrator for further troubleshooting.

The administrator noticed the abnormal behavior of the three PMs (TPS, Max_Active_ DB_Connection, WindowsCheck_icmp_ping), which are well-known root causes of IIS hanging on the web server. Similar to Anomaly #3, the administrator turned off and restarted the IIS of the webserver to resolve this anomaly. In the case of anomaly #5, the other two PMs (Check_SessionPerSessionSetting and Throughput(BPS)) showed anomalous behavior as there were more users than usual. Table 13 shows the alternative CMCs of the anomaly #5 case.

**Target SPI Anomaly #6**. Anomaly #6 occurred on 14 July 2022 from 13:02 to 13:16. Anomaly #6 was detected after the response time exceeded the threshold. However, the visualization result did not display any unusual flaw. Furthermore, testing of the SVPN authentication by the service administrator did not yield any unusual result, and no error was detected in the connection to the SVPN webpage. Even though no further actions were taken, all the PMs and the response time returned to their normal ranges. Thus, we categorized them as unknown events.

**Reducing search space and search time for finding CMs**. The ACMA remarkably reduces the search time for finding the root causes of performance anomalies by focusing on the PMs that have a greater influence on the target SPI. As shown in Table 14, we measured the search space and average search time of the ACMA in the ENS (SVPN and Samsung uReady) mentioned in Section 3.1. Recall that ACMA only considers 62 vital PMs out of all the 191 PMs. Table 14 demonstrates that ACMA achieves a noticeable reduction in search space size and time through an efficient root cause analysis using the hybrid approach.

Specifically, there are total $2^{191}-1$ combinations of PMs in the naive top-down searching approach. However, the ACMA reduces the search space by selecting ten primary CMCs from among all the vital PMs by using a bottom-up approach and selects alternative CMs from among the remaining vital PMs by applying a top-down approach. Consequently, the average search time for finding the root causes of performance anomalies decrease significantly compared to that of the naive approach (6.5 days). More specifically, when the root causes exist in the primary CMCs, we can resolve the performance anomaly within one minute. Moreover, the ACMA is expected to derive the alternative CMCs within seven minutes, when the root causes are not in the primary CMs. Such a prompt detection and analysis of performance anomalies at an early stage allows the administrator to improve the reliability and continuity of the Web service hosted on a multitier system.

### 5.3. Evaluation of the Accuracy of Anomaly Detection

While the main objective of the ACMA is to bridge the gap between anomaly detection and root cause analysis, analyzing the root causes of an anomaly should be preceded by the detection of the target SPI anomalies. To evaluate the anomaly detection accuracy of the proposed framework, we performed experiments with the following settings.

#### 5.3.1. Experimental Settings

**Evaluation metrics** Precision and recall are widely used to measure the accuracy of a classification problem. Note that the ACMA compares the estimated target SPI value with the incoming target SPI value to determine whether a performance anomaly exists in the system. Obtaining a positive anomaly detection result whenever an incoming target SPI value exceeds the threshold could result in numerous false alarms. To prevent such a situation, we set the detection interval to three minutes. Thus, the detector determines whether a performance anomaly occurs given the average of the target SPI values collected over three minutes. Precision is defined as a measure of relevant predictions by the classifier ($\frac{\mathrm{True}\;\mathrm{positive}}{\mathrm{True}\;\mathrm{positive}\;+\;\mathrm{False}\;\mathrm{positive}}$), whereas recall provides us a how many genuinely relevant results are returned ($\frac{\mathrm{True}\;\mathrm{positive}}{\mathrm{True}\;\mathrm{positive}+\;\mathrm{False}\;\mathrm{negative}}$). While high values of both precision and recall are desirable, recall is more important than precision in our study, as missing one performance anomaly might result in a catastrophic loss in enterprise business.

**Comparison method.** Opprentice [40] extracts results of underlying statistical detectors (e.g., moving average, Holt-Winters, and SVD) as features and trains a random forest classifier with these features to detect anomalies ultimately. The random forest classifier in Opprentice needs user feedback (i.e., supervised learning), which requires the operator to manually label whether the past value of the target SPI is anomalous or not. Note that the ACMA’s random forest classifier estimates the future value of the target SPI based on the values of the vital PMs as features (i.e., unsupervised learning), which implies that the labeling process is unnecessary. Accordingly, we had to label the collected values of the target SPI one by one to compare the performance of ACMA with that of Opprentice. Specifically, we have collected 87,840 data points of the response time of SVPN over the past two months. It took an average of five seconds to label individual data points, and a total of 122 h were required to process all data points. For the underlying detectors of Opprentice, we leveraged ACMA’s detector (Section 4.4).

#### 5.3.2. Experimental Results

**Figure 25:**
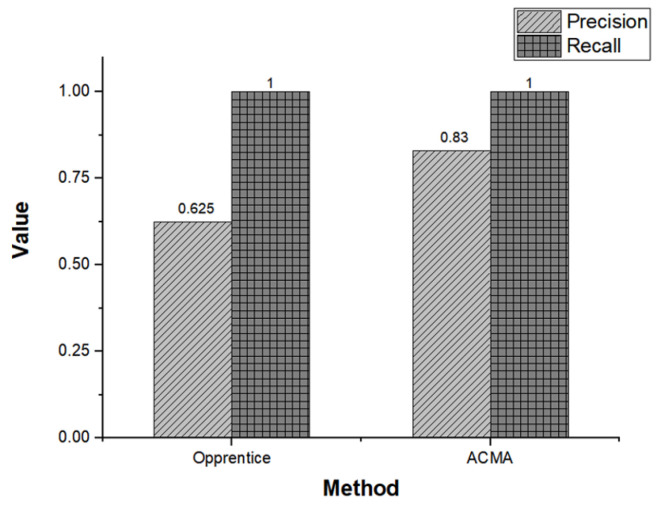
Accuracy of anomaly detection of Opprentice and ACMA.

Figure 25 shows the anomaly detection accuracy of ACMA and Opprentice. Evidently, both ACMA and Opprentice have the same recall performance. Specifically, Opprentice also detected all the anomaly cases in Section 5.2, which implies that both ACMA and Oppretice could notify the administrator about the anomalous activities that could result in catastrophic loss in the business service. However, ACMA showed better performance in precision than Oppentice. As described in Section 5.2, while ACMA misjudged only one case (occurred at 14 July 2022 13:04), Opprentice yielded three more false positive cases (occurred at 20 June 2022 09:06, 19 July 2022 09:36, and 28 July 2022 17:24). Due to the bottom-up approach, ACMA is more efficient than Opprentice in terms of precision. To be specific, Opprentice only examines the variation of the target SPI without considering the influence of various PMs. Classifying only individual data points of target SPI thereby yields a higher number of falsely detected anomalies.

Although these false positive cases do not incur direct problems in business service, generating numerous false alarms places a large burden on the administrator because then they have to check the operational service status whenever the alarm occurs. Moreover, Opprentice requires a data labeling process to train the underlying pattern of the target SPI. When the target SPI is changed, additional labeling work is expected, which increases the burden on the system administrator. In summary, we demonstrate that the ACMA can provide a better business system reliability by detecting anomalous patterns in the target SPI in advance. Furthermore, we consider that the deployment of ACMA is a reasonable choice as it produces fewer false alarms and lightens the labeling burden on the administrator.

### 5.4. Evaluation of the Quality of Root Cause Analysis

#### 5.4.1. Experimental Settings

Since recommending the relevant items to the users is essential for the recommendation system, we can consider the ACMA as a root cause recommendation system. In this section, we have tested the ranking quality of root cause analysis with the following settings.

**Evaluation metrics.** To evaluate the quality of the CMs provided by our model, we used normalized discounted cumulative gain (NDCG@k), which is a widely used metric for comparing the performance of recommendation systems. Because recommending the relevant items to the users is crucial for a recommendation system, the ACMA can be considered as a root cause recommendation system. Formally, NDCG@k is defined as follows:

$$NDCG@k=\frac{DCG@k}{IDCG@k}$$

First, DCG@k accounts for the positions of the top-*k* recommended items by assigning the higher relevance score at the top position ranks as follows:

$$DCG@k=\sum_{i=1}^{k}\frac{rel_{i}}{log_{2}\left(i+1\right)}$$

Here, $rel_{i}$ refers to the granted relevance score of the item at position *i*. IDCG@k is the value of DCG@k, when the items are ideally recommended to the users. In our study, we requested the administrator to provide ranks of the primary and alternative CMCs (a total of 20 PMs) for each anomaly case and assumed these provided ranks as the ideal results. The relevance score for each PM is defined as:

$$rel_{i}=\left\{\begin{array}{cc} \frac{1}{rank_{i}} & if\;rank_{i}\leq 20\\ 0 & otherwise \end{array}\right.$$

where $rank_{i}$ is the graded rank of the item at position *i* by model. Briefly, NDCG@k is in the range $\left[0,1\right]$ and a higher value means the model gives results to the ideal case. In our experiments, we evaluate the NDCG@k by varying the value of the *k* ($k\in \left\{5,10,15,20\right\}$).

**Comparison method** Peiris et al. [4] proposed PAD, a tool to analyze and track the PMs in multiserver distributed systems. PAD assists system administrators’ ability to analyze performance anomalies through various components including data visualization and automated correlation analysis. Specifically, the correlation analysis component in PAD provides the PMs that have a Pearson or Spearman coefficient value greater than the threshold as the metrics responsible for the performance anomalies. Since calculating the Pearson and Spearman coefficients for all the PMs is a time-consuming process, we assume that PAD also knows the vital PMs drawn by the ACMA. Thus, PAD gives rank on the 62 vital PMs based on the Pearson and Spearman coefficients in each anomaly case.

#### 5.4.2. Experimental Results

Table 15 shows the quality of the PMs derived using the root cause analysis feature of the ACMA and PAD for the five anomaly cases. For each anomaly case, we observed that NDCG@k increases as *k* increases for all the methods. Note that in the case of PAD, the correlation analysis is conducted on 62 vital PMs instead of all the PMs. Even though PAD has a reduced search space for analyzing the root causes, the maximum NDCG@k of the PAD is 0.5. The ACMA significantly outperforms PAD in terms of NDCG@k for all the anomaly cases. This result indicates that it is beneficial to select the primary and alternative CMCs by sorting the vital PMs according to their impact on the variation of the target SPI. Furthermore, the NDCG@k of the ACMA is consistently close to 1 (i.e., the ideal case), which implies that the ACMA always provides a good starting point to the administrator to troubleshoot the performance anomaly. In summary, our ACMA model is more suitable for the task of root cause analysis because of its ability to sort the PMs according to their relevance to the target SPI.

## 6. Related Work

Based on study reported in [1], the ACMA falls into performance anomaly detection and bottleneck identification (PADBI) systems, where the bottleneck indicates the potential root causes of the performance anomalies. The two challenges that PADBI systems faces are: (1) how to detect performance anomalies and (2) how to identify the root causes of the detected anomalies. Generally, a method that mainly focuses on the first challenge can be classified as a performance anomaly detection (PAD) system, whereas the work that deals with the second challenge is a performance bottleneck identification (PBI) system.

**Performance anomaly detection (PAD).** In various domains, anomaly detection is employed as the process of identifying outliers or deviations compared to the normal range, such as fraud detection for credit cards and bank transactions. In this study, we focused on the problem of finding performance issues in large-scale systems. Many studies attempt to detect performance anomalies by using various statistical and machine learning algorithms on time series PMs. Ren et al. [2] leveraged the spectral residual model from the computer vision domain. Their main idea was that saliency detection in images can be translated into a problem of detecting anomalies in time-series business metrics. They further combined their spectral residual model with a convolutional neural network (CNN) to improve the accuracy of the time-series anomaly detection. Xu et al. [41] proposed DONUT, which uses a variational auto-encoder (VAE) to model the normal pattern of a time series SPI. DONUT considers the SPI measurements that do not follow the normal pattern learned by VAE as performance anomalies. Shipmon et al. [42] proposed various neural network models and anomaly detection rules to find the sustained anomalies in time series SPI. They discovered that anomaly detection rules are more important than the anomaly detection model, when the time series has no periodicity. Vallis et al. [43] focused on the detection of long-term anomalies. Because of the predominant trend and seasonal components in the time series, they applied the time series decomposition method to extract trend and seasonal components. Then, they detected the performance anomalies in the time series where the trend and seasonal components are removed. Laptev et al. [3] proposed EGADS, which is a generic and scalable framework that can combine the collection of existing anomaly detection models. Similar to EGADS, Liu et al. [40] proposed Opprentice that uses a set of the existing anomaly detectors as anomaly feature extractors. Kim et al. [44] proposed a simulation-based automatic monitoring system (SAM) that could monitor the enterprise system from the users’ viewpoint. The SAM could locate anomalies that could not be detected by the existing monitoring systems. However, the SAM had limits in searching for the root causes in real time.

In summary, researchers have focused on the performance anomaly detection problem from various perspectives and constructed many algorithms. While these methods can extract the outliers from time-series SPI data, their ability to explain the performance anomaly causes is severely limited. As a result, top-down approaches that first identify the anomaly points using performance anomaly detection techniques and subsequently explore the PMs often fail in facilitating a prompt problem diagnosis.

**Root cause analysis.** Root cause analysis is a process of finding the reasons for an observed performance anomaly (i.e., software bugs, system components). To date, several techniques have been demonstrated to assess the potential root cause using machine learning techniques by identifying the relationship between the system metrics. DRACO [45] and DISTALYZER [46] use a supervised learning approach for the analysis of performance logs. In these approaches, data labeling with system logs is required, when the system shows an abnormal behavior, to evaluate the relationship between the PMs and the target SPI. However, since system failure cases are rare, obtaining such labeled data is not always possible. Roy et al. [47] proposed PerfAugur for explaining the potential root cause of cloud services. PerfAugur first organizes a relation table that contains a set of system metrics and a target SPI as attributes and then examines the relationship between them using robust statistics. However, such an approach is more likely to provide a minor root cause if the primary root cause is outside the relation table. Similarly, Peiris et al. [4] proposed a PAD system that detects the potential root causes of a performance anomaly in a distributed system based on a correlation analysis performed using the Pearson and Spearman correlation coefficient. Due to the exponential number of combinations between the PMs, PAD is computationally infeasible, because the Pearson/Spearman correlation coefficient evaluates a pairwise linear relationship. Some systems that detect the root causes of a performance anomaly in the applications hosted in Infrastructure-as-as service [48] or Platform-as-a-Service [49] cloud. These works track events within platform-level instrumentation and thus can provide only limited insights into the system components.

To summarize, researchers have studied the root cause analysis problem from various environments and proposed many statistical methods. Most of them focused on identifying the root causes after a problem occurs. However, this strategy often fails to provide the primary factors that affect the target SPI the most. Table 16 classifies the recent reported methods similar to ACMA. In addition, we compared the program execution time for finding CMs. The program execution time is averaged over five runs. We observed that ACMA took less time to find the CMs than PAD. Specifically, ACMA took an average of 152 s to find the CMs, and PAD took an average of 1287 s. Thus, ACMA is more suitable for analyzing and identifying the root causes of performance anomalies in enterprise services.

## 7. Conclusions

Here, we proposed a system—ACMA—that automatically detects the performance anomalies and analyzes their CMs in real time. The main objective of the ACMA is to provide helpful guidance on performance anomalies to the administrators and minimize the time required to analyze the root causes of a performance anomaly running on a multitier architecture. Despite being important, integration of both anomaly detection and root cause analysis is a considerable challenge because of the search space among all the combinations of possible causes. To solve this problem, we identified a total of 191 PMs that are widely used in a multitier Web system environment. Then, we extracted 62 vital PMs using the statistical methodology to narrow the search space. Based on the 62 vital PMs, we proposed a random-forest-based regression model to extract the primary CMCs that strongly influence the target SPI of the service.

When a target SPI anomaly occurs, primary CMs are selected from among the top-*k* primary CMCs and reported to the administrator within a second. Furthermore, the ACMA prepares alternative CMCs from the remaining vital PMs, except from the primary CMCs, in case that the root cause may not exist in the primary CMs. These alternative CMs are selected from the among alternative CMCs and reported to the manager within minutes. By incorporating various statistical methods, the ACMA can adapt to any multitier Web system and enable it to perform real-time anomaly detection with root cause analysis on any target SPI and PM.

Our experimental results obtained from a real-world business service demonstrated that the ACMA could detect any performance anomaly (irrespective of intended or unintended anomalies) and immediately provide their CMs. Moreover, the actual root causes of all the unintended anomalies existed in the primary and alternative CMs. The experimental results showed that the ACMA facilitated a more extensive and comprehensive analysis of the abnormal behavior of multitier systems, and this feature is anticipated to enhance the reliability of enterprise services.

## Figures and Tables

**Table 1:** Search space and average time for finding the CMs of a performance anomaly by a naive approach.

ENS	Number of PMs				Total PMs	Search Space for Finding CMs	Average Time for Finding CMs
	Applications	DB	OSs	Network			
ENS1	57	31	58	45	191	${}_{191}C_{20}$	6.5 days
ENS2	41	29	57	59	186	${}_{186}C_{20}$	5 days

**Table 2:** Vital PMs of the application domain (15 vital PMs).

No.	Performance Metric	Descriptions	Threshold	Monitoring Cycle
1	Process_CPU_Usage (%)	CPU utilization of the process being monitored	value <90	1 s
2	Process_Mem_Usage (MB)	Memory utilization of the process being monitored (e.g., JVM process memory usage for Java)	value <2048	1 s
3	Heap Memory Usage (%)	Measuring the allocating size of the Java Virtual Machine(JVM) heap memory area	value <95	1 s
4	Current_Thread	The number of created threads	value <100	1 s
5	GC_Time (ms)	Garbage collection (GC) time	value <1000	1 s
6	GC Activity (%)	Percentage of garbage collection usage time	value <3	1 s
7	Active users	The number of users actually executing the transaction	value <5000	1 min
8	TPS	Transactions per second	value <3000	1 s
9	CPU_Time_per_Transaction	CPU time used during a transaction	value <4000	1 min
10	Active_SQL	Number of the SQL statement	value <100	1 s
11	SQL_per_Transaction	Total number of SQL calls divided by the number of transactions	value <1000	1 min
12	External_Call_Time (ms)	Average execution time of one external call	value <5000	1 min
13	Fetch_Number	Sum of numbers measured by fetch	value < 10,000	1 min
14	Fetch_Time (ms)	Average execution time per fetch	value < 10,000	1 min
15	Max_Active_DB_Connection	Maximum number of active DB connections	value <30	1 s

**Table 3:** Vital PMs of the OS domain (13 vital PMs).

No.	Performance Metric	Descriptions	Threshold	Monitoring Cycle
1	WindowsCheck_hostname	Hostname consistency check	1 or more non-response	1 min
2	WindowsCheck_icmp_ping	Internet Control Message Protocol (ICMP) ping status to a Windows server	1 or more non-response	1 min
3	WindowsCheck_wcpu_processorTime	Percentage of time the processor runs non-idle threads	90%	1 min
4	WindowsCheck_weventlog_usage	The ratio of the used capacity to the total capacity of the event log	96%	5 min
5	WindowsCheck_wld_free_space_pct	Percentage of free space available on the logical disk drive	15% or less	5 min
6	WindowsCheck_wmem_usage	The ratio of the used capacity to the total memory capacity (Windows)	90%	1 min
7	Linux_check_sys	Percentage of CPU time spent in user mode(usr) + Percentage of CPU time spent in system mode(sys)	90%	1 min
8	Linux_check_cpu_usage	CPU time spent on system tasks	80%	1 min
9	Linux_check_user_memory_usage	The ratio of the used capacity to the total memory capacity (Linux)	90%	1 min
10	Linux_check_file_pct	Percentage of the filesystem in use	90%	1 min
11	Linux_check_inode_pct	Percentage of inodes allocated	90%	1 min
12	Linux_check_usage	Percentage of capacity in use to total swap capacity	70%	1 min
13	Linux_check_icmp_ping	Internet Control Message Protocol (ICMP) ping status to a Linux server	1 or more non-response	1 min

**Table 4:** Vital PMs of the network domain (12 vital PMs).

No.	Performance Metric	Descriptions	Threshold	Monitoring Cycle
1	nw_cpu	CPU utilization	80%	1 min
2	nw_memory	Memory utilization	80%	1 min
3	nw_RTT	Ping check whether the device is accessible	1 ms per 100 km	1 s
4	Link_status	Check for port link status	Up/Down	1 s
5	Throughput (BPS)	Bandwidth—Min/Avg/Max (Bits per second, BPS)	80%	1 min
6	Throughput (PPS)	Packet throughput—Min/Avg/Max (Packets per second, PPS)	80%	1 min
7	CPS	Session throughput—Min/Avg/Max (Character per second, CPS)	90%	1 min
8	Error	Packet(In/Out) error rate	1%	1 min
9	Discard	Checking for discarded packets due to insufficient buffer	1%	1 min
10	Broadcast	Received broadcast packet monitoring	0.50%	1 min
11	CRC	The number of packets with abnormal CRC value through packet checksum (Cyclic redundancy check, CRC)	1%	1 min
12	Collision	Number of packets retransmitted due to Ethernet collision	1%	1 min

**Table 5:** Vital PMs of the DB domain (22 vital PMs).

No.	Performance Metric	Descriptions	Threshold	Monitoring Cycle
1	RedologFileStatus	Redo log file status (0: Normal, 100: Warning)	value = 100	1440 min
2	CountOfDFPerDBfiles	The number of current datafiles/DB_FILES * 100	80 ≤ value < 90	1440 min
3	TableSpaceFreeSpace	Table space available space—DATAFILE ≤ Threshold	value ≤ 1024 M	60 min
4	lib_pin_cnt	The number of pin events	1 ≤ value < 5	1 min
5	local_dbconn_status	Connection check in the DB server (1: Normal, 0: Failure)	-	1 min
6	waitingLock	Lock request time (max)	5 ≤ value < 20	1 min
7	SessionlnLongLock	Lock holding time (max)	5 ≤ value < 20	1 min
8	TableSpaceFreeSpacePct	The free percentage of tablespace	5 ≤ value < 10	60 min
9	fra_usage	Flashback recovery area (FRA) usage rate	80 ≤ value < 90	1 min
10	check_cpu_idle	CPU Idle time	5 s or less	1 min
11	check_cpu_runqueue	Number of processes in run queue	More than 10	1 min
12	check_cpu_util	Usage active on CPU	90%	1 min
13	check_fs_used_pct	Percentage of the filesystem in use	85%	5 min
14	check_inode_used_pct	Percentage of inodes in use	90%	5 min
15	check_mem_usage	The ratio of used capacity to total memory capacity	90%	1 min
16	check_CountOfDfPerDbfiles	(The number of current datafiles/DB_FILES) * 100	90%	5 min
17	check_LockPerLockSetting	(Current DML_LOCKS / DML_LOCKS) * 100	90%	5 min
18	check_ProcessPerProcessSetting	(Current Processes / Processes) * 100	90%	5 min
19	check_SessionPerSessionSetting	(Current Sessions / Sessions) * 100	90%	5 min
20	check_InvalidRollbackSegment	The status of the rollback segment	80%	5 min
21	check_proc_count	The average number of currently running processes	When 0	1 min
22	check_proc_cpuutil	CPU rate of the process at present	90%	1 min

**Table 6:** Hardware information of the ACMA servers.

ACMA Servers	Descriptions	
ACMA web server	OS	Windows server 2012 R2 standard edition
	CPU	4Core
	Memory	16 GB
	Disk	200 GB
	Model	Lenovo X3650 M5
ACMA analytics server	OS	CentOS 7.4.1708
	CPU	8Core
	Memory	16 GB
	Disk	400 GB
	Model	Lenovo X3650 M5
ACMA search server	OS	CentOS 7.4.1708
	CPU	8Core
	Memory	16 GB
	Disk	500 GB
	Model	Lenovo X3650 M5
ACMA collection server	OS	CentOS 7.4.1708
	CPU	4Core
	Memory	16 GB
	Disk	500 GB
	Model	Lenovo X3650 M5
ACMA DB server	OS	Oracle Linux 7.2
	CPU	16Core
	Memory	64 GB
	Disk	500 GB
	Model	DELL PowerEdge R930
	Oracle Ver.	ORACLE 11g (11.2.0.4)

**Table 7:** Experimental settings.

Conditions	Descriptions	
Study subject	Samsung Virtual Private Network (SVPN)	
Key function	SVPN Authentication (www.samsungvpn.com)	
Target SPI	Response time	
Threshold of target SPI	4 s	
Primary causative metric candidates	Application domain	Transaction Per Second (TPS) Max_Active_DB_Connection Process_CPU_Usage
	OS domain	Windows_check_hostname Windows_check_icmp_ping
	Network domain	Throughput(BPS) error
	DB domain	Check_SessionPerSessionSetting Check_CPU_Util Check_InvaildRollbackSegment

**Table 8:** Summary of the anomaly cases in Section 5.2.

No.	Occurrence Date	Descriptions	Root Cause Location	Provided CMs (Rank)
1	7 June 2022 13:38∼13:48	- SVPN webpage could not be accessed - ID/PW authentication failure	Primary CMs	(Network) Throughput(BPS) (1st) (Application) TPS (2nd) (Application) Max_Active_DB_Connection (3rd) (OS) WindowsCheck_icmp_ping (3rd)
2	13 June 2022 09:04∼09:12	- Slow connection in SVPN webpage - Certification authentication failure	Primary CMs	(Application) TPS (1st) (Network) Throughput(BPS) (1st) (OS) WindowsCheck_icmp_ping (1st) (Network) Error (1st)
3	6 July 2022 10:09∼10:19	- SVPN webpage could not be accessed - ID/PW authentication failure	Primary CMs	(Network) Throughput(BPS) (1st) (Application) Max_Active_DB_Connection (1st) (OS) WindowsCheck_icmp_ping (2nd)
4	17 July 2022 00:02∼00:15	- SVPN webpage could not be accessed	Alternative CMs	(Network) CPS (1st) (Network) Discard (1st) (Network) Throughput(PPS) (1st) (Application) Process_Mem_Usage(MB) (2nd) (OS) WindowsCheck_wcpu_processorTime (2nd) (Application) Current_Thread (2nd) (Network) Collision (2nd) (Application) GC_Time(ms) (2nd) (Network) Error (3rd)
5	29 July 2022 09:08∼09:22	- SVPN webpage could not be accessed - ID/PW authentication failure	Primary CMs	(Application) TPS (1st) (Network) Throughput(BPS) (1st) (DB) check_SessionPerSessionSetting (2nd) (Application) Max_Active_DB_Connection (2nd) (OS) WindowsCheck_icmp_ping (3rd)
6	14 July 2022 13:02∼13:16	- Unknown	None	None

**Table 9:** Alternative CMCs of target SPI anomaly #1.

No.	Performance Metric	Measured Value (Avg.)	Threshold	Monitoring Cycle
1	(OS) WindowsCheck_wmem_usage	67%	90%	1 min
2	(Application) Active_SQL	59	100	1 s
3	(OS) WindowsCheck_weventlog_usage	61%	96%	5 min
4	(Network) Throughput(PPS)	34%	80%	1 min
5	(Network) Collision	0.29%	1%	1 min
6	(DB) WaitingLock	11	20	1 s
7	(DB) SessionInLongLock	7	20	1 s
8	(DB) Lib_pin_cnt	2	5	1 s
9	(Application) Process_Mem_Usage	1178 MB	2048 MB	1 s
10	(Application) Current_Thread	47	100	1 s

**Table 10:** Alternative CMCs of target SPI anomaly #2.

No.	Performance Metric	Measured Value (Avg.)	Threshold	Monitoring Cycle
1	(Network) Throughput(PPS)	82%	80%	1 min
2	(Network) CPS	92%	90%	1 min
3	(Application) Current_Thread	107	100	1 s
4	(Network) Discard	0.9%	1%	1 min
5	(Network) Broadcast	0.37%	0.5%	1 min
6	(Network) Collision	0.86%	1%	1 min
7	(Application) Process_Mem_Usage (MB)	1241 MB	2048 MB	1 s
8	(OS) WindowsCheck_wcpu_processorTime	76%	90%	1 min
9	(Application) CPU_Time_per_Transaction	2174	4000	1 min
10	(Application) Active_SQL	59	100	1 s

**Table 11:** Alternative CMCs of target SPI anomaly #3.

No.	Performance Metric	Measured Value (Avg.)	Threshold	Monitoring Cycle
1	(OS) WindowsCheck_wmem_usage	71%	90%	1 min
2	(Network) Throughput(PPS)	52%	80%	1 min
3	(Application) Active_SQL	49	100	1 s
4	(OS) WindowsCheck_weventlog_usage	57%	96%	5 min
5	(Application) Process_Mem_Usage	1096 MB	2048 MB	1 s
6	(Network) Collision	0.21%	1%	1 min
7	(DB) WaitingLock	9	20	1 s
8	(Application) Current_Thread	53	100	1 s
9	(DB) SessionInLongLock	7	20	1 s
10	(DB) Lib_pin_cnt	1	5	1 s

**Table 12:** Alternative CMCs of target SPI anomaly #4.

No.	Performance Metric	Measured Value (Avg.)	Threshold	Monitoring Cycle
1	[✩] (Network) CPS	93%	90%	1 min
2	[○] (Application) Process_Mem_Usage (MB)	2072	2048	1 min
3	[○] (OS) WindowsCheck_wcpu_processorTime	112	100	1 s
4	[✩] (Network) Discard	1.23%	1%	1 min
5	[○](Application) Current_Thread	107	100	1 min
6	[✩] (Network) Throughput(PPS)	87%	80%	1 min
7	[○] (Network) Collision	1.17%	1%	1 s
8	(Application) External_Call_Time(ms)	4078	5000	1 min
9	[○] (Application) GC_Time (ms)	1974	1000	1 min
10	(Application) GC Activity (%)	2	3	1 s

**Table 13:** Alternative CMCs of target SPI anomaly #5.

No.	Performance Metric	Measured Value (Avg.)	Threshold	Monitoring Cycle
1	(OS) WindowsCheck_wmem_usage	67%	90%	1 min
2	(Application) Active_SQL	59	100	1 s
3	(OS) WindowsCheck_weventlog_usage	61%	96%	5 min
4	(Network) Throughput(PPS)	34%	80%	1 min
5	(Network) Collision	0.29%	1%	1 min
6	(DB) WaitingLock	11	20	1 s
7	(DB) SessionInLongLock	7	20	1 s
8	(DB) Lib_pin_cnt	2	5	1 s
9	(Application) Process_Mem_Usage	1178 MB	2048 MB	1 s
10	(Application) Current_Thread	47	100	1 s

**Table 14:** Search space and average time required by the ACMA for finding CMs.

ENS	PMs of a Multitier Web System				Approach for Finding CMs	Search Space for Finding CMs	Avg. Time for Finding CMs
	App. PMs	DB PMs	OS PMs	NW PMs			
ENS	57	31	58	45	Top-down	${}_{191}C_{20}$	6.5 days
ENS via ACMA	15	22	13	12	Primary CMCs → CMs: Bottom-up / Alternative CMCs → CMs: Top-down	Bottom-up: 10 from PME / Top-down: 52 from PMA	If primary CMCs → CMs, then one minute / If alternative CMCs → CMs, then seven minutes

**Table 15:** Quality of the root cause analysis performed by the ACMA and PAD.

Anomaly Case	Anomaly #1		Anomaly #2		Anomaly #3		Anomaly #4		Anomaly #5	
Method	ACMA	PAD	ACMA	PAD	ACMA	PAD	ACMA	PAD	ACMA	PAD
**NDCG@5**	0.88	0.15	0.97	0.15	0.97	0.38	0.83	0.17	1	0.14
**NDCG@10**	0.9	0.34	0.94	0.14	0.98	0.47	0.85	0.31	0.98	0.38
**NDCG@15**	0.9	0.43	0.98	0.3	0.99	0.5	0.86	0.45	0.99	0.38
**NDCG@20**	0.91	0.48	0.98	0.43	0.99	0.49	0.87	0.5	1	0.46

**Table 16:** Comparison between recent literature on anomaly detection and root cause analysis.

Technique	Methodology					Performance		
	Anomaly Detection	Extracting Vital PMs	Finding CMs	Model Flexibility	Providing CMs in Real Time	Search Space for Finding CMs	Avg. Time for Finding CMs	Program Execution Time for Finding CMs
Opprentice [40]	Supported	Unknown	Not supported	Not supported	Not supported	Unknown	Unknown	Unknown
PAD [4]	Supported	Unknown	Supported	Partially supported	Not supported	Large (The framework requires a 1:1 comparison for all metrics in a top-down method)	Long (The framework requires a 1:1 comparison for all metrics in a top-down method)	1287 s
ACMA	Supported	Yes	Supported	Supported	Supported	Bottom-up: 10, Top-down: 52	Primary CM : one min, Alternative CM: seven min	152 s

## Data Availability

By request, the source code will be released after we got the approval of Samsung SDS.
